# Testis-specific protein Y-encoded 1 regulates androgen receptor expression through the MAPK/ERK pathway in male hepatocellular carcinoma

**DOI:** 10.15537/smj.2022.43.10.20220455

**Published:** 2022-10

**Authors:** Zhaolu Lu, Dongmei Yang, Shanzi Qin, Cuiju Mo, Linyan Zhang, Yingying Ou, Shan Li

**Affiliations:** *From the Department of Clinical Laboratory, First Affiliated Hospital of Guangxi Medical University, Guangxi, China.*

**Keywords:** male hepatocellular carcinoma, testis-specific protein Y-encoded 1, androgen receptor, mitogen-activated protein kinase/extracellular signal-related kinase

## Abstract

**Objectives:**

To analyze the mechanism of testis-specific protein Y-encoded 1 (TSPY1) in male hepatocellular carcinoma (HCC).

**Methods:**

This experimental study was carried out at Guangxi Medical University’s First Affiliated Hospital, Guangxi, China, between January 2016 and December 2019. The expression of TSPY1, androgen receptor (AR), messenger ribonucleic acids (mRNAs), and proteins were detected by qRT-PCR and Western blotting. The co-localization and interaction of TSPY1 and AR were observed by immunofluorescence assay and co-immunoprecipitation. Hepatocellular carcinoma cells overexpressing and silencing TSPY1 were constructed, and the expression and phosphorylation levels of TSPY1, AR, and mitogen-activated protein kinases/extracellular signal-regulated kinases (MAPK/ERK) signaling pathway-related key molecules ERK1/2, p38, and JNK were also detected.

**Results:**

The expression levels of TSPY1, AR mRNAs, and proteins were highly positively correlated in HCC cells in different metastatic potentials with a high correlation coefficient of R=0.929 and R=0.884. Testis-specific protein Y-encoded 1 and AR were then co-localized in the nucleus of HCC cells, and TSPY1 and AR can interact with each other. In addition, the expression of AR and phosphorylation of ERK1/2 were enhanced in TSPY1 overexpressed Huh7 cells. They were reduced in HCCLM3 cells with TSPY1 knockdown expression. In addition, in response to blocking MAPK/ERK signaling activity, AR was reduced in expression.

**Conclusion:**

These findings suggested that there was a positive correlation between TSPY1 expression and AR in male HCC cells, and high TSPY1 expression stimulates AR expression, MAPK/ERK signaling pathway may be involved in its mechanism.

**W**orldwide, hepatocellular carcinoma (HCC) is the 6th most common cancer, among men it ranks 5th and among women it ranks 7th.^1^ In most countries, the prevalence is 2-4 times higher in males than in females, demonstrating that gender differences could be linked to the incidence of HCC.^2^

Recently, the testis-specific protein Y-encoded 1 (TSPY1) has been identified as an antigen that accelerates tumor growth. It has also been found that TSPY1 is commonly ectopically expressed in different somatic cancers under pathological conditions, including HCC.^3,4^ Our previous research has discovered that TSPY1 is highly expressed in male HCC and TSPY1 was associated with the proliferation and invasion of the HCC cells.^5^ Despite this, it is unknown what the molecular mechanism of TSPY1 is in male HCC.

Androgen is a male hormone that is increasingly reported in male-dominated HCC. It plays a role in diverse pathological and physiological processes through binding to androgen receptor (AR). The development of HCC cells was discovered to be strengthened by overexpression of AR ex-vivo, in addition to HCC-initiating cells in-vivo.^6^ However, its specific mechanism remains unclear and needs further study.

In the mitogen activated protein kinase (MAPK) cascades, c-Jun N-terminal kinase (JNK) and p38 signaling pathways are mainly related to cell apoptosis, while extracellular signal-regulated kinase 1/2 (ERK1/2) is closely related to cell proliferation and differentiation.^7^ Many studies have shown that p38, JNK, and ERK, are associated with HCC occurrence and development.^8-10^ The activation of TSPY1 and AR in cancer is related to the MAPK pathway, and TSPY1 can increase the expression of the upstream factor Ras in the MAPK/ERK signaling pathway.^11^ Androgen receptor may be a downstream factor of the MAPK/ERK pathway in prostate cancer.^12^ Our previous research has found that cells and tissues of HCC expressed TSPY1 and AR simultaneously, while the function mechanism between them is still unclear.^5^ We hypothesized that TSPY1 can regulate AR expression in male HCC through the MAPK/ERK signaling pathway. Therefore, our study aims to discover the mechanism by which TSPY1 regulates AR expression in male HCC.

## Methods

This experimental study was carried out at Guangxi Medical University’s First Affiliated Hospital, Guangxi, China, between January 2016 and December 2019. We used the search engine PubMed to search for previous relevant studies. Male HCC tissues were acquired from surgical cases at the First Affiliated Hospital of Guangxi Medical University. The Ethical Committee of the First Affiliated Hospital of Guangxi Medical University, Guangxi, China, approved the this study (No. ethical review 2015-KY-NSFC-041), which adhered to the principles of the Declaration of Helsinki.

Male HCC cell lines (HCCLM3, Huh7, HepG2, SMMC7721, MHCC97-L, and MHCC97-H) were obtained from The Cell Bank of Academy of Sciences, Shanghai, China. In an incubator with 5% carbon dioxide (CO_2_) at 37°C, the cells were grown in Dulbecco’s modified Eagle’s medium (DMEM) containing 10% fetal bovine serum (Gibco, Grand Island, NY, USA).

Reverse transcription was used to synthesize complementary DNA (cDNA) from total RNA extracted from cultured cells using TRIzol reagent (Invitrogen Life Technologies, Carlsbad, CA, USA). Real-time quantitative reverse transcription PCR (qRT-PCR) was carried out with the FastStart Universal SYBR Green Master (ROX) kit (Roche Applied Science, Basel, Switzerland) and StepOnePlus™ Real-Time PCR instrument (Bio-Rad, Hercules, Ca, USA). The internal reference was β-actin. We used the 2^-ΔΔCt^ method to get the relative expression of TSPY1 and AR. Table 1 shows a list of primer sequences.

**Table 1:** - List of primers used for quantitative reverse transcription-polymerase chain reaction.

Genes	Primer sequences
TSPY1	Forward: 5’- ATGTTGTTCTTTCGGAGTAACCC -3’ Reverse: 5’- TGAGAAGCCCTGTATTCTGTGAT -3’
AR	Forward: 5’- ACTCCAGGATGCTCTACTTCG -3’ Reverse: 5’- AGGTGCCTCATTCGGACA -3’
β-actin	Forward: 5’- CATGTACGTTGCTATCCAGGC -3’ Reverse: 5’- CTCCTTAATGTCACGCACGAT -3’

TSPY1: testis-specific Y-encoded protein 1, AR: androgen receptor, β-actin: Beta-actin

Sodium dodecyl sulfate-polyacrylamide gel (SDS-PAGE) electrophoresis was carried out on the total protein extracted from the cells. The bands were transferred to polyvinylidene fluoride membrane (Millipore, Bedford, MA, USA). The membrane was sealed with 5% BSA (Beyotime Biotechnology, Shanghai, China) at room temperature for one hour, and then incubated overnight with primary antibodies (TSPY1=1:500, AR=1:1000, ERK1/2=1:1000, p38=1:1000, JNK=1:1000, and β-actin=1:10,000; Abcam, Cambridge, UK) at 4°C. After the wash, the secondary horseradish peroxidase-conjugated antibody was diluted at 1:10,000 (Cell Signaling Technology, Boston, USA) and a one-hour incubation was carried out with the membrane at room temperature. The substrate for the enhanced chemiluminescence (Beyotime Biotechnology, Shanghai, China) western blot analysis was then prepared. The Bio-Rad (Hercules, Ca, USA) ChemiDoc™ XRS Gel Doc, a gel imaging system, was used for imaging and the band intensity was represented as a relative ratio.

All frozen sections of HCC tissues were prepared by the Department of Pathology, First Affiliated Hospital of Guangxi Medical University, Guangxi, China. Hepatocellular carcinoma cells were fixed with 4% paraformaldehyde (Solarbio, Beijing, China) and HCC tissues were fixed with 4°C acetone (Solarbio, Beijing, China) then the use of 0.5% Triton X-100 (Solarbio, Beijing, China) to permeate block with 10% goat serum (Solarbio, Beijing, China). Testis-specific protein Y-encoded 1 was incubated with the rabbit anti-human TSPY1 antibody and the goat anti-rabbit fluorescent secondary antibody according to instructions. Androgen receptor was incubated with mouse anti-human AR antibodies and goat anti-mouse fluorescent secondary antibodies (all antibodies are from Abcam, Cambridge, UK) according to instructions. Nuclear staining was carried out with 4′,6-diamidino-2-phenylindole (DAPI) (Beyotime Biotechnology, Shanghai, China). The co-localization was confirmed with a laser confocal microscope (Leica Microsystems, Wetzlar, Germany).

The instructions of the immunoprecipitation kit (Takara Bioengineering, Dalian, China) were followed. In brief, the proteins from the cells were immunoprecipitated by the rabbit anti-human AR antibody, and the resultant immune complexes were subjected to SDS-PAGE electrophoresis. After membrane blocking, the rabbit anti-human TSPY1 antibody and rabbit anti-human AR antibody (all antibodies are from Abcam, Cambridge, UK) were used for Western blot analysis. Experimental design negative control group.

Overexpression and silencing of TSPY1 in stable transgenic cell lines Gikein Co., Ltd (Shanghai, China) constructed a lentivirus vector for the overexpression of TSPY1 and a lentivirus vector for TSPY1-short-hairpin RNA (shRNA). The overexpression and silencing TSPY1 construction vectors were transfected into Huh7 and HCCLM3 cells. Thereafter, 1 ml of enhanced infection collusion (Gikein Co., Ltd, Shanghai, China), containing polybrene and virus stock, was added to each well. After mixing well, the cells were incubated at 37°C and 5% CO_2_, and the control group was cultured with the same amount of serum-free medium. After 8-12 hours, the supernatant was discarded and serum containing complete DMEM was added to the culture. After 3-4 days, the transfection efficiency was detected by a fluorescence microscope. The protein and mRNA expression of TSPY1 was estimated by western blot and qRT-PCR analyses.

### Statistical analysis

The analysis of the data was carried out using the Statistical Package for the Social Sciences, version 20.0 (IBM Corp., Armonk, NY, USA). Pearson’s product-moment correlation was used for correlation analysis. Two groups were compared with a T-test. Comparing between the groups was carried out by one-way analysis of variance (ANOVA). Statistical significance was determined by a *p*-value of <0.05.

## Results

**Figure 1:**
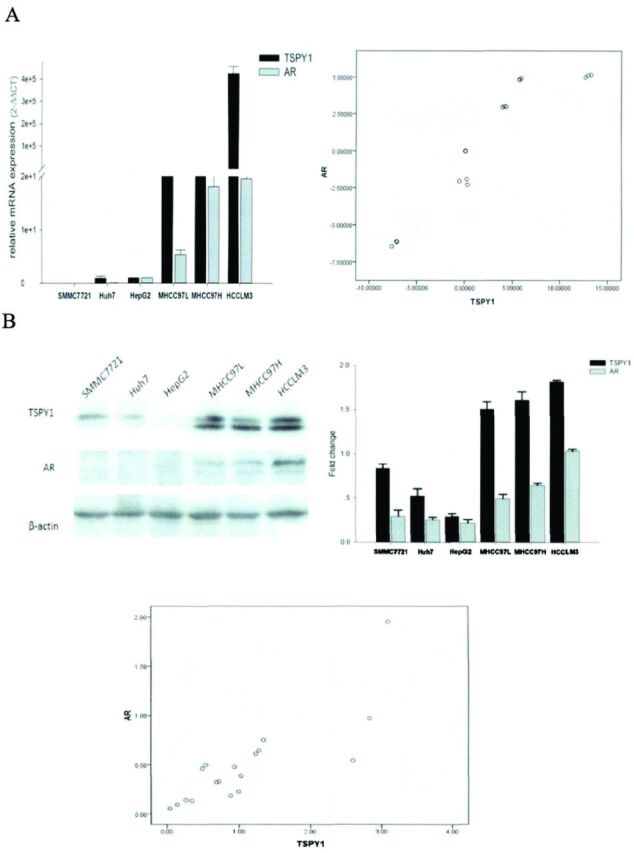
- Expression levels of testis-specific Y-encoded protein 1 (TSPY1) and androgen receptor (AR) messenger ribonucleic acid (mRNA) and protein in hepatocellular carcinoma (HCC) cells. **A**) mRNA expressions levels of the TSPY1 and AR in HCC cells and the mRNA levels correlation analysis scatterplot between TSPY1 and AR (*p*<0.05); **B**) the Western blot bands for TSPY1 protein and AR protein in HCC cells; the histogram of gray value of Western blot bands for each protein and the protein levels correlation analysis scatterplot between TSPY1 and AR (*p*<0.05).

The expression levels of TSPY1 and AR were positively correlated in male HCC cells. Real-time quantitative reverse transcription PCR and western blot analyses were used to estimate the expression levels of TSPY1 and AR from 6 Y chromosome-positive HCC cells with different metastatic potentials and Pearson’s correlation test was used to determine the relationship between TSPY1 and AR (Table 2). In the study, it was found that TSPY1 and AR expression levels were low in HepG2, SMMC7721, and Huh7 cells with no or low metastatic potential, while high in MHCC97-L, MHCC97-H, and HCCLM3 cells with high metastatic potential and the correlation coefficient R was 0.929 for mRNA level (*p*=0.025, *p*<0.05; Figure 1A). Similarly, the expression level of TSPY1 and AR protein achieved a consistent result as mRNA with a high correlation coefficient R value of 0.884 (*p*=0.023, *p*<0.05; Figure 1B). These results indicated that the expression levels of TSPY1 and AR were positively correlated in male HCC cells and may be due to the metastatic potential of HCC cells.

**Table 2:** - The clinical and pathological features of hepatocellular carcinoma tissues.

Features	Hepatocellular carcinoma				
Gender	Male				
Age (years)	39	40	60	77	53
BCLC	B	B	C	A	A
T stage	2	3	2	1	1
N stage	0	1	1	0	0
M stage	0	0	0	0	0
Tumor size	≤5 cm	≤5 cm	≤5 cm	≤5 cm	≤5 cm
HBV DNA (IU/ml)	1.93×10^6^	3.25×10^3^	<5.00×10^2^	<5.00×10^2^	<5.00×10^2^
Hepatitis B surface Ag	Positive	Positive	Positive	Positive	Positive
Serum AFP (ng/ml)	3198.75	6316.99	60.22	33.04	1434.1
PIVKA (ug/L)	1520.7	7010.49	687.56	222.55	552.24

BCLC: Barcelona clinic liver cancer, T stage: the size and extent of the main tumor, N stage: the number of nearby lymph nodes that have cancer, M stage: whether the cancer has metastasized, HBV: hepatitis B virus, AFP: alpha-fetoprotein, PIVKA: protein induced by vitamin K absence

**Figure 2:**
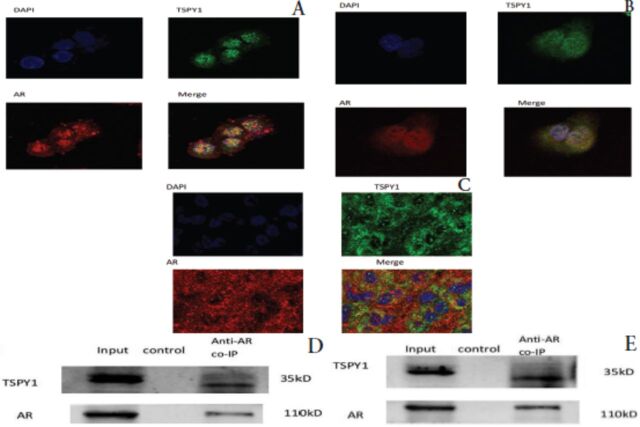
- The co-localization and interaction of testis-specific Y-encoded protein 1 (TSPY1) and androgen receptor (AR). **A**) Co-localization of TSPY1 and AR immunofluorescence in MHCC-97H hepatocellular carcinoma (HCC) cells (400x); **B**) co-localization of TSPY1 and AR immunofluorescence in HCCLM3 HCC cells (400x); **C**) co-localization of TSPY1 and AR immunofluorescence in male HCC tissues (400x); **D**) Western blot detection of TSPY1 protein and AR protein in anti-AR co-immunoprecipitation (Co-IP) in MHCC-97H liver cancer cells; **E**) Western blot detection of TSPY1 protein and AR protein in anti-AR Co-IP in HCCLM3 liver cancer cells (input is the same amount of total protein; control was the negative control; anti-AR Co-IP was the protein sample obtained by immunoprecipitation with rabbit anti-human AR antibody).

Testis-specific protein Y-encoded 1 and AR co-localized and interacted in male HCC cells and tissues. We used immunofluorescence to detect co-localization between TSPY1 and AR in male HCC cells and tissues. Images were obtained using a laser confocal microscope. Testis-specific protein Y-encoded 1 was labeled green, while AR was labeled red, and both were distributed diffusely in the cytoplasm and nucleus. 4′,6-diamidino-2-phenylindole was used to stain the nuclei blue. Testis-specific protein Y-encoded 1 and AR showed obvious co-localization in the nucleus, as seen in yellow (Figure 2A-C). To detect the interaction between TSPY1 and AR in male HCC cells, the total proteins of MHCC-97H and HCCLM3 were extracted for co-immunoprecipitation. Western blot analysis detected the presence of TSPY1 and AR proteins in the co-immunoprecipitation complex, indicating the interaction between TSPY1 and AR (Figure 2D&E).

**Figure 3:**
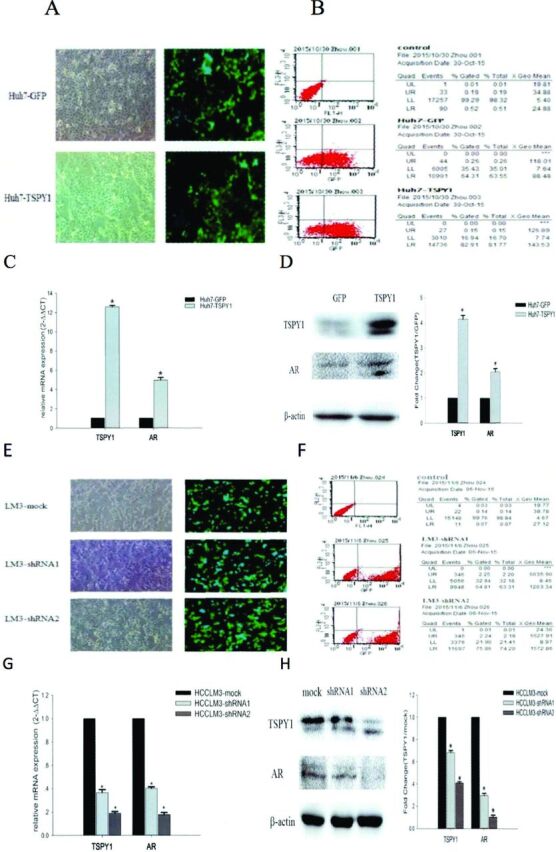
- The role of overexpression and silencing of testis-specific Y-encoded protein 1 (TSPY1) on androgen receptor (AR) regulation in HCC cells. **A**) The fluorescence figure of Huh7 cells over-expressing TSPY1 (100X); **B**) the flow cytometry was used to detect transfection efficiency in the TSPY1 over-expressing Huh7 cells; **C**) effects of over expression of TSPY1 on AR in Huh7 cells: the expression levels of TSPY1 and AR genes (^*^compared with the control group [GFP], *p*<0.05); **D**) AR and TSPY1 protein expression levels in Huh7 cells over-expressing TSPY1 and histogram of gray value of Western blot bands (^*^compared with the GFP group, *p*<0.05); **E**) fluorescence figure of HCCLM3 cells knockdown TSPY1 (100X); **F**) the flow cytometry was used to detect transfection efficiency in the TSPY1 knockdown HCCLM3 cells; **G**) effects of silencing TSPY1 on the regulation of AR in HCCLM3: messenger ribonucleic acid expression levels of TSPY1 and AR in HCCLM3 cells in silencing TSPY1 (^*^compared with the control group [mock], *p*<0.05); **H**) AR and TSPY1 protein expression levels in HCCLM3 cells silencing TSPY1 and histogram of gray value of Western blot bands (^*^compared with the mock group, *p*<0.05).

The overexpression and silencing expression of TSPY1 could regulate the expression of AR in HCC cells. To investigate the impact of TSPY1 in the expression of AR, 2 HCC cells, Huh7, and HCCLM3 were transfected with TSPY1 cDNA lentivirus and TSPY1-shRNA lentivirus to overexpress and silence the expression of TSPY1. The transfection efficiency of Huh7 was 81.77% (Figure 3A&B) and HCCLM3 was 74.2% (Figure 3E&F). The results of western blot and qRT-PCR further confirmed that the expression levels of TSPY1 mRNAs and proteins were clearly increased in Huh7 cell transfection with TSPY1 cDNA (*p*=0.034, *p*<0.05; Figure 3C&D), while the expression of TSPY1 was down-regulated in LM3 cell transformation with TSPY1-shRNA (*p*=0.024, *p*<0.05; Figure 3G&H). In addition, our findings showed that AR mRNAs and proteins were more abundant in Huh7 cells overexpressing TSPY1 than in control cells (*p*=0.036, *p*<0.05; Figure 3C&D). While the expression levels of AR were lower in HCCLM3 cells with TSPY1 silenced than that in the control group (*p*=0.027, *p*<0.05; Figure 3G&H), these results suggested that AR expression can be positively regulated in HCC cells by overexpressing or silencing TSPY1 expression.

**Figure 4:**
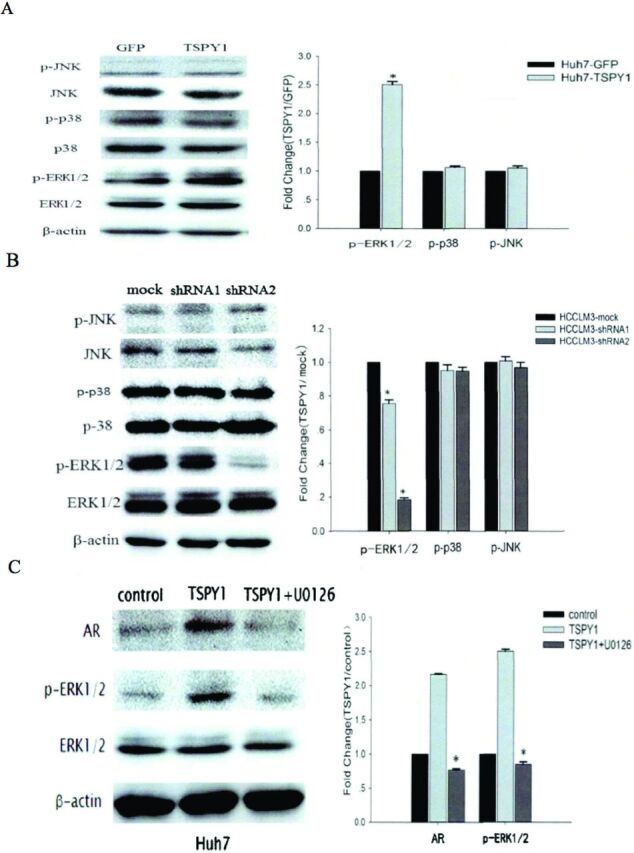
- The mechanism by which testis-specific Y-encoded protein 1 (TSPY1) regulates androgen receptor (AR) expression in male HCC. **A**) Key molecules expression levels of mitogen activated protein kinase (MAPK) signaling pathways and their phosphorylation levels in Huh7 cells over-expressing TSPY1 and the histogram of gray value of Western blot bands (^*^compared with the control group [GFP], *p*<0.05); **B**) key molecules expression levels of MAPK signaling pathways and their phosphorylation levels in HCCLM3 cells silencing TSPY1 and the histogram of gray value of Western blot bands (^*^compared with the control group [mock group], *p*<0.05); **C**) expression levels of AR and P-ERK1/2 in Huh7 cells after inhibition of MAPK/ERK signaling pathway and the histogram of gray value of Western blot bands for each protein (^*^compared with the TSPY1 group, *p*<0.05).

Overexpression of TSPY1 can up-regulate the AR level by promoting activation of the MAPK/ERK signaling pathway. To validate the influence of TSPY1 on MAPK signaling pathway, the phosphorylation levels of the key molecules of MAPK pathway (such as ERK1/2, p38, and JNK) were analyzed using western blot. As shown in Figure 4A, the phosphorylation level of ERK1/2 increased in Huh7 cells overexpressing TSPY1 (*p*=0.036, *p*<0.05), while the phosphorylation levels of JNK and p38 showed no obvious change (*p*=0.075). In contrast, the phosphorylation level of ERK1/2 was lower in HCCLM3 cells where the expression of TSPY1 was silenced compared to the control group (*p*=0.025, *p*<0.05), while the phosphorylation levels of p38 and JNK showed no significant change (*p*=0.068; Figure 4B). According to the above results, overexpression of TSPY1 can promote activation of the MAPK/ERK signaling pathway. We used U0126 to inhibit the activation of the MAPK/ERK pathway in TSPY1 over-expressed Huh7 cells. As shown in Figure 4C, the increase of phospho-ERK1/2 protein level was blocked markedly in U0126 treated TSPY1 over-expressed Huh7 cell (*p*=0.021, *p*<0.05). We next examined whether the expression of AR was changed after the signaling pathway was blocked by U0126 in TSPY1 over-expressed Huh7 cells. Western blot analyses showed that the expression of AR protein decreased significantly in the TSPY1 over-expressed Huh7 cells treated with U0126 (*p*=0.024, *p*<0.05; Figure 4C). It indicated that the expression of AR seems to be regulated by the MAPK/ERK signaling pathway. In other words, overexpression of TSPY1 can update the AR level by promoting activation of the MAPK/ERK signaling pathway.

## Discussion

In recent years, the molecular mechanisms of HCC have been widely studied. However, there are still many unknowns regarding HCC, especially in terms of geography, ethnicity, and gender. Among men, HCC incidence is higher, and there is a correlation between male androgens, androgen receptors, and in HCC tissues, AR expression is abnormally high.^13-15^ We previously found TSPY1 to be highly expressed in hepatitis B virus-positive male HCC tissues, but low in normal male and female HCC tissues. Testis specific protein Y-linked 1 overexpression influences HCC cell growth and invasion, which is linked to androgen levels.^5^ These data suggested that TSPY1 and AR play an important role in male HCC.

Based on our findings, we concluded that gene and protein expression levels of TSPY1 and AR tended to increase in hepatoma cells from non-metastatic or low-metastatic potential to high-metastatic potential. The expression trend of AR in HCC cells with different metastatic potential agrees with the results of AoJ et al,^16^ and the correlation analysis found that the 2 were highly positively correlated; that provide a reliable theoretical basis that TSPY1 regulates the expression of AR. To verify whether TSPY1 regulates AR, TSPY1 was overexpressed and silenced in Huh7 and HCCLM3 cells, based on the results, compared to HCCLM3, we found Huh7 cells expressed more AR mRNA and protein, while HCCLM3 cells expressed less AR. Moreover, immunofluorescence co-localization assay also showed that both TSPY1 and AR co-localize in the cytoplasm and nucleus, predominantly in the nucleus. Based on these results, we hypothesized that TSPY1 binds AR in the cytoplasm, which facilitates AR entry into the nucleus, after which it binds to specific regions of DNA sequences and activates transcription of target genes. Co-immunoprecipitation experiments showed the presence of TSPY1 and AR proteins in the precipitated immune complexes by western blot, indicating an interaction between TSPY1 and AR.

In HCC, activation of MAPK/ERK signaling is frequent. Studies have shown that the mRNAs of RAF and ERK are overexpressed of HCC patients.^17^ Researchers have discovered that cancers are closely related to the Ras pathway, and the major downstream factors of Ras signaling included ERK1/2.^18,19^ Testis specific protein Y-linked 1 was found to contribute to the up-regulation p-ERK1/2 in RAS signaling pathway.^3^ Therefore, TSPY1 may enhance the phosphorylation level of key molecules (such as Ras) by activating the related regulatory factors of the ERK1/2 signaling pathway, thereby activating this pathway. As a result of this study, it was found that the phosphorylation level of ERK1/2 was significantly increased in cells overexpressing TSPY1, while the level of ERK1/2 phosphorylation was significantly lower in cells with silenced TSPY1 expression. These findings confirmed that TSPY1 regulates the phosphorylation level of the ERK1/2 pathway. Previous studies have found that AR is an important downstream molecule of the MAPK/ERK signaling pathway in prostate cancer. Inhibition of the ERK1/2 pathway leads to low expression of AR.^20-22^ Based on the results of this study, TSPY1 can regulate AR expression through MAPK/ERK signaling. In addition to ERK1/2 phosphorylation, the expression of AR increased or decreased with TSPY1. Inhibition of the ERK1/2 signaling pathway also reduced AR expression in Huh7 cells overexpressing TSPY1. These results confirmed that TSPY1 regulates AR expression through the MAPK/ERK pathway in male hepatoma cells.

### Study limitations

We only studied the changes of signaling pathway and AR expression after overexpressing TSPY1 but not silencing TSPY1. The second limitation is that we also did not study how the MAPK/ERK pathway regulates AR expression. The third point is that we only did in vitro experiments, not in vivo experiments. Therefore, our prospects are to further investigate the regulatory relationship between TSPY1 and AR and establish a mouse model to verify their relationship in vivo.

In conclusion, this study preliminarily explored the mechanism of TSPY1-mediated regulation of AR expression in male HCC and found that TSPY1 may activate the MAPK/ERK pathway to regulate AR expression. The findings provide a scientific basis for further study of the molecular mechanisms that cause and develop male HCC.
